# Leveraging a multidimensional linguistic analysis of constructed responses produced by college readers

**DOI:** 10.3389/fpsyg.2022.936162

**Published:** 2022-08-10

**Authors:** Joseph P. Magliano, Lauren Flynn, Daniel P. Feller, Kathryn S. McCarthy, Danielle S. McNamara, Laura Allen

**Affiliations:** ^1^Department of Learning Sciences, Georgia State University, Atlanta, GA, United States; ^2^Department of Educational Psychology, The University of Minnesota, Minneapolis, MN, United States; ^3^Department of Psychology, Arizona State University, Tempe, AZ, United States

**Keywords:** comprehension, college readers, constructed responses, natural language processing, individual differences

## Abstract

The goal of this study was to assess the relationships between computational approaches to analyzing constructed responses made during reading and individual differences in the foundational skills of reading in college readers. We also explored if these relationships were consistent across texts and samples collected at different institutions and texts. The study made use of archival data that involved college participants who produced typed constructed responses under thinking aloud instructions reading history and science texts. They also took assessments of vocabulary knowledge and proficiency in comprehension. The protocols were analyzed to assess two different ways to determine their cohesion. One approach involved assessing how readers established connections with themselves (i.e., to other constructed responses they produced). The other approach involved assessing connections between the constructed responses and the texts that were read. Additionally, the comparisons were made by assessing both lexical (i.e., word matching) and semantic (i.e., high dimensional semantic spaces) comparisons. The result showed that both approaches for analyzing cohesion and making the comparisons were correlated with vocabulary knowledge and comprehension proficiency. The implications of the results for theory and practice are discussed.

## Introduction

Reading is a fundamental skill for college students. Unfortunately, research suggests that many college students struggle to learn from texts, particularly for the complex expository texts that are often required reading in college classes. It has been estimated that 75% of community college and 50% of four-year students are not adequately prepared for the reading literacy demands of their college coursework (ACT, 2006; Baer et al., 2006; Holschuh and Paulson, 2013). This is in part because the *reading for understanding* that is required for college courses involves a coordination of a variety of processes and strategies that support the construction of an elaborated and coherent mental model for a text (McNamara and Magliano, 2009; Perfetti and Stafura, 2014). Theories of comprehension emphasize that establishing coherence in the mental model is essential for understanding, which means that readers are able to represent how content conveyed in a text is semantically related and how relevant background knowledge is integrated into the mental model (e.g., Kintsch, 1988; Graesser et al., 1994). This representation arises from an interplay of conceptually lower (decoding, lexical access, sentence processes) and higher level processes (inference strategies; Cromley and Azevedo, 2007; Perfetti and Stafura, 2014; Magliano et al., 2020). Foundational skills of reading support processing the printed word, which provides input for the higher order processes that support the construction of a coherent mental model. Some college readers struggle with the foundational skills associated with word reading (Ari, 2016; Halldórsdóttir et al., 2016; Magliano et al., in press; Kopatich et al., 2022), others may be proficient readers, but do not engage in higher order comprehension strategies that support comprehension (Magliano and Millis, 2003; Kopatich et al., 2022), some struggle with both (Magliano et al., in press; Kopatich et al., 2022). These findings suggest that providing support for these different types of struggling college students requires a deeper understanding of the relations between foundational reading skills and comprehension outcomes and better means of evaluating students’ different strengths and weaknesses (Magliano and Millis, 2003; Magliano et al., 2011; Perin, 2020).

One approach for studying college readers is through the analysis of constructed responses. Constructed responses produced during reading (e.g., think-alouds) have provided insights into the strengths and challenges of college readers (Magliano and Millis, 2003; Magliano et al., 2011, 2020, in press; Cromley and Wills, 2016; Feller et al., 2020). These responses reveal a variety of strategies that support the construction of a mental model (Pressley and Afflerbach, 1995; Trabasso and Magliano, 1996; McNamara, 2004; Magliano et al., 2011) and are correlated with performance on standardized measures or the foundational skills of reading in college readers (Magliano and Millis, 2003; Magliano et al., 2011, 2020). As such, constructed responses have been useful in the study of underprepared college readers.

Traditionally, constructed responses have been analyzed through expert judgments (i.e., annotation) in which raters use a codebook or scoring rubric to identify different processes and strategies evident in the constructed responses (e.g., Pressley and Afflerbach, 1995; Chi, 1997; McNamara, 2004; Rapp et al., 2007). More recently, there has been a growing interest in leveraging computational analyses to quantify linguistic features of the constructed responses. Natural language processing approaches are sensitive to subtle features of the readers’ language that are informative of individual differences in reading proficiency and potentially indicative of sense making (Allen et al., 2016). The present study examines how theoretically motivated and computationally-derived indices of *cohesion* can be used to explore aspects of coherence-building and the extent to which these measures are related to college students’ foundational reading skills.

### Foundational skills, inference strategies, and comprehension

Reading for understanding involves coordination of a set of skills that support the process of reading and the construction of a coherent mental model (Cromley and Azevedo, 2007; Perfetti and Stafura, 2014; Kopatich et al., 2018). In the context of the present study, we make a distinction between foundational skills and coherence building strategies. Foundational skills involve word, sentence, and discourse level processes that enable readers to construct propositional representations of text sentences that reflect the content that was explicitly conveyed in the text and how propositions are referentially related to one another (Perfetti and Stafura, 2014). Processing words starts the process of activating knowledge needed to build a mental model that is the basis of comprehension, and as such word recognition facilitates processing that supports comprehension (e.g., Perfetti and Stafura, 2014). As such, in the present study we explored the relationship between constructed responses and the foundational skills associated with vocabulary knowledge and discourse comprehension.

Text comprehension involves building a coherent mental model for a text (Graesser et al., 1994). The mental model consists of a network of propositions that reflect content explicitly conveyed and knowledge-based inferences (i.e., inferences generated based on the knowledge that readers activate during reading; Kintsch, 1988). Coherence reflects the relationships that are established between the propositions. That is, coherence building strategies establish how propositions corresponding to sentences are related to one another or integrate relevant background knowledge into the mental model. These strategies range from resolving anaphora (i.e., identifying the references to pronouns) to generating explanatory inferences that specify how propositions are connected (Graesser et al., 1994). Theories of comprehension make a distinction between local and global coherence (McNamara and Magliano, 2009). Specifically, local coherence is established when connections are made between adjacent sentences, whereas global coherence is established when readers establish connections across sentences that are more distally apart. Comprehension requires establishing coherence at both local and global levels (e.g., Graesser et al., 1994).

Contemporary models of reading literacy assume that there are relations between foundational skills of reading and coherence building strategies (Cromley and Azevedo, 2007; Perfetti and Stafura, 2014; Kopatich et al., 2018; Magliano et al., 2020). Some research focuses on testing moderational relationships (e.g., Magliano and Millis, 2003), whereas others focus on testing the presence of mediational relationships (e.g., Cromley and Azevedo, 2007; Kopatich et al., 2018). However, these approaches generally focus on mapping foundational skills to the specific strategies that readers employ during reading (e.g., the frequency that they engage in bridging or paraphrasing). In the present study, we take a different approach by examining the *cohesion* of the constructed responses at multiple levels. Our argument is that cohesion indices can serve as proxies for coherence-building, as they indicate the amount and type of connections that readers are making while reading. Thus, our goal was to examine how these cohesion measures related to individual differences in vocabulary knowledge and comprehension proficiency. Our long-term goal is to leverage these findings for future development of research and interventions directed at using these approaches to better understand the strengths and challenges of college readers.

Importantly, some contemporary frameworks of literacy assume that reading can vary across contexts (Snow, 2002; Britt et al., 2018). Context can involve place and time where the literacy activity takes place, the texts that students read, and the nature of the tasks. The present study therefore takes advantage of archival data that affords the exploration of the extent to which estimated relations between the linguistic features of constructed responses and foundational skills of reading vary are stable across institutions (i.e., community colleges and a university) and texts (i.e., science and history texts).

### Analyzing constructed responses

The use of human judgments or expert ratings of constructed responses generated during reading was popularized in the late 1980s and early 1990s (e.g., Ericsson and Simon, 1993; Pressley and Afflerbach, 1995; Trabasso and Magliano, 1996). Since then, it has continued to be a fruitful approach for the study of text comprehension processes (e.g., Rapp et al., 2007; Goldman et al., 2012; Cromley and Wills, 2016). Over the past two decades, advances in natural language processing (NLP) techniques have afforded researchers increased opportunities to examine constructed responses along multiple dimensions (e.g., Magliano et al., 2002, 2011; Magliano and Millis, 2003; McNamara et al., 2006; Millis et al., 2007; Allen et al., 2016). These advances have not only made it easier and more efficient to evaluate constructed responses, but they have also made it possible to calculate linguistic features of the responses that would be difficult for human raters to identify.

Computational analyses of constructed responses are often focused on exploring the extent that readers engage in coherence building strategies (Allen et al., 2016; Magliano and Millis, 2003; Magliano et al., 2011). However, there are several different aims or goals within this body of work. For example, some research uses linguistic analysis to measure the *quality* of the responses (McNamara et al., 2004) while others have aimed to identify the specific *strategies* (e.g., paraphrasing, bridging) that readers are employing (Magliano et al., 2011). Relevant to the current research study, researchers have also examined coherence building by modeling the connections that readers make across various pieces of the text (Allen et al., 2015, 2016), and how the relationship between these various features to comprehension outcomes and individual differences (Allen et al., 2015, 2016). When constructing a coherent mental model of a text, a reader must generate links or connections between concepts. Cohesion is a measure (construct) that captures the ways in which ideas are linked (McNamara et al., 2010). Cohesion analyses involve assessing the degree to which constructed responses overlap with each other. In other words, this approach examines the degree to which readers establish connections across the content within their constructed responses. Recent research suggests that automated analyses of cohesion can be used to assess these connections and can predict individual differences in knowledge and skills as well as learning from the text (Allen et al., 2015, 2016). Cohesion isn’t a single thing; it can be assessed in a variety of ways in service of understanding coherence building. Our intent is not to pit different approaches against one another, but rather to more systematically examine how different aspects of cohesion might be used to better understand the coherence-building processes engaged by students, and particularly struggling students.

One aspect of cohesion reflects “what” is being connected (see Table 1). Cohesion can be measured using *lexical overlap* – when readers repeat the same content words (nouns, verbs, adverbs, adjectives) from sentence to sentence or echo the words from the text they are reading. Lexical comparisons are made with word matching algorithms that detect if the same words are used in the units of language that are being compared (e.g., Magliano et al., 2011). While researchers may create a dictionary of synonyms to assist these comparisons of constructed responses to the text, others have chosen to constrain lexical comparisons to the exact words in the text (e.g., Magliano et al., 2011). Our approach examines whether words overlap across the constructed responses that readers produced. For example, consider a student who is reading a text about red blood cells. For the first response, the student states, “Red blood cells are a necessity for the body. They bring carbon dioxide to the cells of the body. The body then turns the oxygen into carbon dioxide. The red blood cells take the carbon dioxide and have it removed.” Later in the text, the student states, “Anemia is a condition where not enough oxygen gets into the body. Anemia can make a person feel tired and weak. One time, my doctor told me I was anemic and it made me feel really tired. I guess, anemia must have something to do with problems with your red blood cells. Is blood a cell?” Here, we can see that there are multiple words that the student repeats across constructed responses, such as “body” and “cells.” Lexical overlap measures will count the degree to which the reader uses these same words across the responses.

**TABLE 1:** A summary of the computational approaches for analyzing constructed responses.

Computational approach	Grain size	Lexical	Semantic
Cohesion overlap	Local	Adjacent responses: lexical overlap	Adjacent responses: Word2vec
	Distal	Adjacent (+2) responses: lexical overlap	Adjacent (+2) responses: Word2vec
Source overlap	Source	Proportion of unique words (types) that overlap with source	All responses to source text: Word2vec

In addition to specific words or close synonyms, another way of assessing cohesion is through *semantic overlap.* Computationally, semantic overlap is calculated through the use of high dimensional semantic spaces (e.g., Latent Semantic Analysis; Landauer and Dumais, 1997; Word2vec; Mikolov et al., 2013). High dimensional semantic spaces are statistical approaches for representing knowledge about words and the world on the basis of a large corpus of texts. Semantic spaces are constructed by analyzing a corpus of thousands or even millions of texts to count how frequently different words co-occur with the assumption that the meaning of words are linked to the words for which they tend to co-occur (Landauer and Dumais, 1997; Burgess et al., 1998; Jones et al., 2006). That is, a set of words share meaning to the extent that occur in similar contexts. For example, “banana” and “apple” share meaning because they are used in similar contexts and thus are closer in semantic space, whereas “banana” and “stapler” are less semantically related. With respect to the example above, a student may not explicitly use the word “body” in their responses; however, if they are talking about their arms or other parts of their body, these responses are still likely to be highly semantically related. The general recommendation is that both lexical and semantic overlap are important for the computational assessment of constructed responses (McNamara et al., 2007; McNamara, 2011; Magliano and Graesser, 2012).

One way of evaluating the quality of a reader’s mental model and coherence is to evaluate *source overlap* (see Table 1). Source overlap reflects the extent to which the reader represents the content words (lexical) or ideas (semantic) that are presented in the text. On the one hand, a reader who is representing too little of the text content may be struggling to identify key ideas or may be off-task. On the other hand, a reader who is representing too much of the text may be including only what is explicit in the text and not making critical inferences to more fully elaborate the mental model. Of course, some words and/or ideas are going to be more important for the construction of an accurate or complete mental model. Thus, one approach to measuring source text overlap is to identify a dictionary of key terms that appear in the source text and calculate how many of those terms appear in the students’ responses (e.g., Millis et al., 2007).

In addition to exploring overlap between source and response, researchers have begun to more deeply examine aspects of cohesion *within* a reader’s response called *cohesion overlap* (see Table 1). Such approaches were initially developed in tools such as Coh-Metrix (Graesser et al., 2004; McNamara et al., 2004) that were designed to calculate indices of cohesion to measure text complexity. These analyses assume that texts that are less cohesive are more complex as they require the reader to generate more inferences in order to maintain coherence (McNamara and Kintsch, 1996). In recent years, researchers have been exploring the extent to which these cohesion indices can be used to infer the coherence of a reader’s mental model. For example, Allen et al. (2016) asked readers to generate constructed responses while reading a text about natural selection. NLP techniques were then used to calculate the cohesion of these responses. The results indicated that cohesion values were predictive of text-specific prior knowledge and comprehension. Of relevance to the current study and its focus on coherence-building, measures can be calculated at multiple grain sizes such that you can examine if readers generate connections to the constructed responses that were produced immediately prior as well as ones produced earlier during reading.

Measures of *local overlap* examine how words or ideas are connected across two adjacent sentences in the students’ response. The underlying assumption is that local overlap suggests that the reader is engaging in coherence-building processes that are supporting the maintenance of local coherence. While local cohesion is important, it is also critical for readers to be making connections across the larger discourse context. Thus, measures of overlap can be calculated at different grain sizes to examine how readers generate connections across constructed responses produced earlier during reading. The distinction between local and distal can be operationalized in multiple ways. In some cases, local overlap is defined as overlap to only the adjacent sentence and anything beyond that reflects distal or even global overlap (e.g., Magliano et al., 2011). In the current study, we examine two primary grain sizes: overlap for adjacent sentences (local cohesion) in the constructed responses and overlap of two adjacent constructed responses (more distal cohesion). There are numerous grain sizes at which cohesion can be measured; here, we chose two that represent relatively local and distal connections that readers may be making while reading.

### Overview of study and research questions

Our overarching aim was to use a multidimensional linguistic analysis of think aloud responses made during reading to examine how students’ constructed responses reflect individual differences in foundational skills of reading in college readers. We also explored if these relationships were consistent across texts and samples collected at different institutions. This study uses archival data from Magliano et al. (2020) in which college students produced typed “think aloud” responses while reading expository texts (i.e., science and history texts). This sample contains college readers from two- and four-year institutions wherein some of the students were designated as needing additional support in reading literacy. The participants produced constructed responses while reading a history and a science text. They also were administered an assessment of their proficiencies in the components of foundational reading skills. We conducted the study to address the following two research questions:

*RQ1: How do readers’ foundational reading skills (vocabulary, reading comprehension) relate to the cohesion of constructed responses?*

*RQ2: To what extent do these relations vary across contexts (i.e., text and sample)?*

Based on prior research, we hypothesized that linguistic analyses of overlap with the text and cohesion of constructed responses would be positively correlated with proficiencies in vocabulary and comprehension (Allen et al., 2015, 2016; Feller et al., 2020). We did not specify *a priori* hypotheses regarding the extent that these relations vary across institutions and texts.

## Method

### Statement of ethics compliance

The research presented in this article was reviewed by an institutional human subjects compliance board and all participants signed an informed consent form before their participation.

### Corpus (data set)

This study is a secondary analysis of data collected in Magliano et al. (2020). The data set includes 560 students from a large, 4-year institution in the Midwest (*n* = 263), a community college in the Southwest (*n* = 265), and a community college in the Northeast (*n* = 32). In the current analysis, we include the 495 students *who completed both measures (described below) and provided demographic information.* Of the participants that reported their gender (*n* = 392), about 62% identified as female. Of those who selected an age range (*n* = 373), about 90% were between 18 and 22 years old (range = 18–22 to 50–53). Additionally, of the students that reported whether or not English was their first language (*n* = 392), approximately 76% reported English as their first language.

### Measures

#### Foundational reading skills

Foundational skills of reading were measured using the Study Aid and Reading Assessment (SARA; Sabatini et al., 2015, 2019). In the current study, we focused on two subtests: vocabulary and reading comprehension. The vocabulary subtest involved presenting target words and participants were asked to select a synonym or topically related word from three choices. The range of possible scores was 0–38. Previously reported reliability estimates range from 0.72 to 0.81 for high school students (Sabatini et al., 2019). In the Reading Comprehension subtest, participants read passages and answered multiple-choice questions about each passage that involved understanding main ideas, locating important details, and drawing inferences. The range of possible scores was 0–22. Previously reported reliability estimates range from 0.80 to 0.85 (Sabatini et al., 2019).

#### Reading strategy assessment tool

The Reading Strategy Assessment Tool (RSAT; Magliano et al., 2011) was used to collect the think aloud protocols. Participants produced typed constructed responses while reading two texts: Power of Erosion and Louis the XVI and the French Revolution. The Power of Erosion is an earth science text that describes the basic processes underlying erosion and its role in forming geological features. It contains 316 words, 22 sentences, and 5 paragraphs and has a Flesch-Kincaid grade level of 9.9. Participants produced think aloud responses after 7 sentences that were chosen by Magliano et al. (2011) because they afforded bridging and elaborative inference strategies based on a theoretical analysis of the texts. Louis the XVI and the French Revolution is a history text that makes the case that Louis the XVI has been misunderstood with respect to the events that lead up to the French Revolution. It contains 366 words, 19 sentences, and 4 paragraphs and has a Flesch-Kincaid grade level of 11.7. Participants produced think-aloud responses after 6 sentences.

### Data processing

To examine the cohesion of the constructed responses generated by participants, we first aggregated participants’ responses for each text and separated them by a paragraph break (Allen et al., 2015). That is, each participant’s data was combined into a single science response comprising the seven constructed responses generated while reading that text and a history response (comprising the six constructed responses generated during that text), such that each response was represented as its own paragraph.

We then cleaned the responses by correcting misspellings and editing contractions to be two separate words. NLP tools were then used to calculate the lexical and semantic cohesion of the responses at three different grain sizes, resulting in six cohesion indices (see Table 1). For the calculation of all variables except the lexical, source-based indices, we relied on the Tool for the Automatic Analysis of Text Cohesion (TAACO; Crossley et al., 2016), a freely available NLP tool that provides automated measures related to text cohesion. TAACO allows users to calculate indices of local and global cohesion for a given text, ranging from the repetition of words used in the text to the semantic similarity of the paragraphs. For the *lexical, source overlap indices*, we calculated these through simple counts of overlapping lemmas (i.e., *run*, *runs*, and *running* would be considered overlapping because they share the same lemma, *run*) from the source text. For the *local and distal lexical cohesion* indices, we calculated overlap across adjacent responses or adjacent + 2 responses, respectively. For these measures, the overlap was operationalized as the number of words that overlapped with the next (adjacent) or next two (adjacent + 2) responses. These were normalized by the total number of responses that the students produced. These measures provided an indication of lexical cohesion and were intended to represent the times that students were linking ideas and concepts across their constructed responses. For the lexical cohesion indices, the values could be as low as 0 (indicating no lemma overlap) with no explicit cap on the upper end of the range because it is based on the number of lemmas that overlap and thus the length of their responses. In the context of our data, however, the upper end of the range was 20. Here, higher values indicate that more lemmas are repeated across the constructed responses that students generated.

For the *lexical source overlap* measures, we calculated lexical overlap as the proportion of unique words from the text (i.e., the number of word types) that were in the constructed responses to avoid bias from a single keyword in the source text. In other words, we wanted to examine the breadth of topics that students drew on from the source text rather than simply counting the number of times they referenced the primary topic of the text (e.g., erosion). This index was therefore intended to represent the degree to which students were drawing on the concepts from the source text in the responses they generated.

*Semantic cohesion* was calculated at local (adjacent responses), distal (adjacent + 2 responses), and source text levels. For all three of these indices, semantic cohesion was measured using TAACO (Crossley et al., 2016). TAACO relies on Word2vec to calculate semantic similarity at multiple levels. Similar to LSA (Landauer and Dumais, 1997), Word2vec is an NLP technique for calculating semantic similarity by estimating associations between words using a large corpus of texts (Mikolov et al., 2013). Words in the texts are treated as vectors that are decomposed mathematically and similarity is computed using a cosine value (ranging from 0 to 1). The higher the cosine value, the more strongly two words are semantically related. In comparison to LSA, Word2vec uses of multiple-layered neural network (whereas LSA applies singular value decomposition) and it leverages an increased contextual window that considers words both before and after a target word. After the Word2vec model has been trained, it can detect words that are considered highly similar based on the contexts in which they tend to occur. Therefore, while lexical overlap captures explicit overlap of one’s words across responses (i.e., repeating the word “erosion”), semantic overlap affords for less overt word overlap, instead giving way for semantically-similar word overlap (i.e., using words with similar meanings such as “revolution” and “war”). The only difference in the calculation of the three indices related to the segments of text that were being compared: for local and distal indices, we calculated the average Word2vec cosine value between adjacent and adjacent + 2 responses, respectively. For the source overlap index, we calculated similarity with the text that students read.

Given that the students generated constructed responses for two texts, we aggregated the NLP indices at the participant-level for all analyses with the exception of the exploratory genre analysis in research question two.

### Data accessibility

The data and R scripts can be accessed online on Open Science Framework (https://osf.io/6fthp/).

## Results

Students’ aggregated constructed responses contained an average of 85.97 words (SD = 48.11), ranging from 10 to 492. Correlation analyses were conducted to examine relations between readers’ proficiencies in foundational skills (i.e., vocabulary and reading comprehension) and linguistic features of constructed responses. Given that variables were not normally distributed, Spearman’s Rho was used to examine the relations among variables (Chen and Popovich, 2002). Descriptive statistics and correlations are presented in Table 1. Participants on average scored 69.6% (*M* = 26.46; SD = 6.08) and 55.5% (*M* = 12.20; SD = 4.29) on the vocabulary and reading comprehension components of SARA, respectively. These scores indicate that, overall, students had relatively strong skills but that these abilities varied considerably across our sample. Performance on these two proficiency components were significantly correlated (*r* = 0.63, *p* < 0.001).

*RQ1: How do readers’ foundational reading skills (vocabulary, reading comprehension) relate to the cohesion of constructed responses?*

To address our first research question, the strength of the relations among vocabulary, reading comprehension, and linguistic features of constructed responses was examined. The correlations among foundational reading skills are presented in Table 2.

**TABLE 2:** Overall descriptive statistics and Spearman correlations.

		SARA score correlation			
		
Index type	Overlap index	*M*	SD	Vocabulary	Reading comprehension
Descriptive	Word count	85.97	48.11	0.41***	0.42***
	Adj. response overlap	2.49	1.69	0.26***	0.30***
Lexical cohesion	Adj. 2-response overlap	3.78	2.33	0.28***	0.31***
	Prop. of type overlap	0.10	0.06	0.28***	0.31***
	Adj. response Word2vec	0.78	0.05	0.22***	0.26***
Semantic cohesion	Adj. 2-response Word2vec	0.79	0.06	0.19***	0.20***
	Source similarity Word2vec	0.64	0.15	0.21***	0.21***
	Vocabulary	26.46	6.08	–	0.63***
	Reading comprehension	12.20	4.28	–	–

*Indicates p < 0.05. **Indicates p < 0.01. ***Indicates p < 0.001.

Word count was a significant positive predictor of vocabulary and reading comprehension scores. This is not surprising, as it is likely that students who are more engaged in reading the texts will produce longer responses on average. Additionally, both vocabulary and reading comprehension scores were significantly, positively related to markers of cohesion associated with word overlap at both local and distal grain sizes. This suggests that participants with higher word knowledge and better comprehension skills were more likely to generate constructed responses that connected to the prior responses they generated.

Similarly, vocabulary and reading comprehension scores were significantly, positively correlated with indices of semantic overlap at both window sizes. These findings are similar to those for lexical overlap and indicate that higher world knowledge and comprehension skills are associated with strong connections at both the word and semantic levels. Measures of semantic overlap differ from measures of word overlap in that they reflect that readers are not only making explicit connections to specific words in their response, but they are also establishing connections by generating responses that refer to similar concepts. Finally, vocabulary and reading comprehension scores were both positively correlated with cohesion indices that reflected connections made to the source text. In particular, overlap at the word and semantic levels were correlated with these scores, a significant, positive correlation was found with type overlap with the source text (*r* = 0.28, *p* < 0.001). This suggests that those with stronger vocabulary skills typically make more explicit lexical connections in their constructed responses than those with weaker vocabulary skills. Finally, reading comprehension scores were positively associated with lemma type overlap with the source text (*r* = 0.31, *p* < 0.001), suggesting that students with higher comprehension scores were more likely to use a greater proportion of lemma types (unique lemmas) from the source text.

A multiple linear regression was used to examine the predictive power of the indices when taken together and controlling for the overall length of the aggregated constructed responses (see Table 3 for detailed model information). Due to multicollinearity (Variance Inflation Factor >10), one index was not included in the model: Adj. 2-Response Overlap. The overall model significantly predicted vocabulary scores (*R*^2^ = 0.18, *F*(6,488) = 17.92, *p* < 0.001). Importantly, while word count was the strongest predictor of vocabulary scores, all of the remaining indices except lexical overlap to the source remained significant. As such, these metrics account for unique variance in vocabulary knowledge even when accounting for the length of the responses. A similar regression examined the ability of these indices to predict reading comprehension scores (see Table 4 for detailed model information). Due to multicollinearity (Variance Inflation Factor > 10), one index was not included in the model: Adj. 2-Response Overlap. The overall model significantly predicted reading comprehension scores (*R*^2^ = 0.21, *F*(6,488) = 21.15, *p* < 0.001). The results of this analysis were similar to those of the vocabulary analyses – namely all indices except lexical overlap to the source text were significant in the model, even after controlling for response length. This indicates that both vocabulary knowledge and reading comprehension are related to cohesion metrics at multiple levels; however, the results also suggest that these variables may not be particularly strong at discriminating amongst these two individual difference measures. As expected, word count was the strongest predictor in the model; however, the majority of the cohesion indices remained significant when this measure was entered in the models. For both models, the source overlap indices were the weakest, indicating that the connections that students established within their constructed responses were more predictive than the connections they made to the source text.

**TABLE 3:** Linear model predicting SARA vocabulary scores.

Predictor	Estimate	*df*	Std. error	*t*	*p*	Partial η^2^
(Intercept)	14.85	1	4.24	3.50	<0.001	
Word count	0.11	1	0.01	7.92	<0.001	0.11
Adj. response overlap	−2.05	1	0.36	−5.70	<0.001	0.06
Prop. of type overlap	−10.32	1	8.71	−1.18	0.237	0.00
Adj. response Word2vec	29.38	1	12.42	2.37	0.018	0.01
Adj. 2-response Word2vec	−24.65	1	9.93	−2.48	0.013	0.01
Source similarity Word2vec	7.81	1	2.60	3.00	0.003	0.02

**TABLE 4:** Linear model predicting SARA comprehension scores.

Predictor	Estimate	*df*	Std. error	*t*	*p*	Partial η^2^
(Intercept)	3.53	1	2.94	1.20	0.23	
Word count	0.07	1	0.01	7.06	<0.001	0.09
Adj. response overlap	−1.16	1	0.25	−4.66	<0.001	0.04
Prop. of type overlap	0.24	1	6.05	0.04	0.968	0
Adj. response Word2vec	29.18	1	8.62	3.39	<0.001	0.02
Adj. 2-response Word2vec	−24.73	1	6.89	−3.59	<0.001	0.03
Source similarity Word2vec	3.99	1	1.81	2.21	0.028	0.01

*RQ2: To what extent do these relations vary across contexts (i.e., text and sample)?*

To address our second research question, we examined the extent to which the relations between cohesion and the SARA scores were stable across text genre (i.e., history, science) and institution (i.e., 2-year, 4-year). Correlations by genre and institution are presented in Tables 5, 6, respectively.

**TABLE 5:** Descriptive statistics and Spearman correlations by genre.

		SARA score correlation			
		
Index type	Overlap index	*M*	SD	Vocabulary	Reading comprehension
				History	
Descriptive	Word count	83.77	48.11	0.41***	0.44***
	Adj. response overlap	2.61	1.99	0.26***	0.31***
Lexical cohesion	Adj. 2-response overlap	3.92	2.70	0.28***	0.31***
	Prop. of type overlap	0.08	0.05	0.23***	0.29***
	Adj. response Word2vec	0.77	0.08	0.19***	0.22***
Semantic cohesion	Adj. 2-response Word2vec	0.77	0.09	0.16***	0.17***
	Source similarity Word2vec	0.56	0.18	0.16***	0.17***
				Science	
Descriptive	Word count	88.17	48.05	0.37***	0.36***
	Adj. response overlap	2.37	1.72	0.23***	0.24***
Lexical cohesion	Adj. 2-response overlap	3.64	2.38	0.24***	0.24***
	Prop. of type overlap	0.12	0.06	0.28***	0.28***
	Adj. response Word2vec	0.79	0.06	0.16**	0.17**
Semantic cohesion	Adj. 2-response Word2vec	0.81	0.07	0.15**	0.13**
	Source similarity Word2vec	0.72	0.17	0.23***	0.23***

*Indicates p < 0.05. **Indicates p < 0.01. ***Indicates p < 0.001.

**TABLE 6:** Descriptive statistics and Spearman correlations by institution.

		SARA score correlation			
		
Cohesion type	Overlap index	*M*	SD	Vocabulary	Reading comprehension
			2-year institution		
Descriptive	Word count	85.99	49.50	0.39***	0.43***
	Adj. response overlap	2.59	1.84	0.24***	0.35***
Lexical cohesion	Adj. 2-response overlap	3.87	2.49	0.28***	0.34***
	Prop. of type overlap	0.11	0.06	0.24***	0.33***
	Adj. response Word2vec	0.78	0.05	0.22***	0.33***
Semantic cohesion	Adj. 2-response Word2vec	0.79	0.05	0.200**	0.26***
	Source similarity Word2vec	0.65	0.16	0.18**	0.32***
			4-year institution		
Descriptive	Word count	85.95	41.76	0.42***	0.41***
	Adj. response overlap	2.40	1.53	0.31***	0.27***
Lexical cohesion	Adj. 2-response overlap	3.69	2.18	0.31***	0.29***
	Prop. of type overlap	0.10	0.05	0.39***	0.32***
	Adj. response Word2vec	0.78	0.06	0.26***	0.22***
Semantic cohesion	Adj. 2-response Word2vec	0.78	0.07	0.22***	0.16*
	Source similarity Word2vec	0.62	0.15	0.32***	0.16**

*Indicates p < 0.05. **Indicates p < 0.01. ***Indicates p < 0.001.

We first examined correlations across the two text genres. While the general pattern of results was largely consistent across genres, the strength of these relations varied. Word count had a stronger relation with both vocabulary and reading comprehension in the history text than the science text. Similarly, the relations among vocabulary, reading comprehension, distal response lexical overlap, local response Word2vec, and distal Word2vec were stronger in the history text than the science text. This suggests that constructed responses for the history text used slightly greater word and semantic overlap from the source text than those corresponding to the science text. Overlap with the source text also appeared to vary by text genre, with a stronger negative relationship between lemma type overlap and vocabulary for the history text than the science text. Conversely, the relation between type overlap and vocabulary, though statistically insignificant, was stronger for the science text than the history text.

In terms of institutional differences, there were various relations that varied in magnitude. In general, measures of both word and semantic overlap (i.e., local response lexical overlap, distal response lexical overlap; local response Word2vec, distal response Word2vec) were more strongly correlated with reading comprehension scores in the 2-year institution than the 4-year institution. Additionally, vocabulary scores were more highly correlated with adjacent response overlap for the 4-year institution than the 2-year institution; however, reading comprehension scores were more highly correlated with adjacent response overlap for 2-year institution than the 4-year institution. Lemma overlap also appeared to vary by institution. There was a significant negative correlation between lemma overlap and vocabulary in the 2-year institution but not the 4-year institution. This suggests that the negative relation between vocabulary and lemma overlap was stronger for participants in the 2-year institution. Lemma type (i.e., unique lemmas) also varied by institution, with a stronger relation between vocabulary and lemma type in the 4-year institution than the 2-year institution.

## Discussion

The purpose of the present study was to explore the relations between college students’ foundational reading skills and their coherence-building processes. We used natural language processing tools to calculate a multidimensional analysis coherence-building strategies based on indices reflecting cohesion. More specifically, we examined cohesion at both lexical and semantic levels and across multiple levels of connection (i.e., local overlap, global overlap, source text overlap). We addressed two research questions. The first research question (How do readers’ foundational reading skills (vocabulary, reading comprehension) relate to the cohesion of constructed responses?) pertained to whether the different approaches were correlated with the foundational skills of vocabulary knowledge and comprehension proficiency. The results of the present study are consistent with prior research that shows that the computational analysis of constructed response associated with coherence building strategies are correlated with individual differences in foundational skills of reading (Magliano and Millis, 2003; Allen et al., 2015, 2016; Feller et al., 2020; Magliano et al., 2020). All six indices of cohesion were positively and significantly correlated with vocabulary and reading comprehension proficiency suggesting that readers with stronger foundational skills in reading were creating more connections as they read. The strongest relations were with the explicit lexical connections made across the larger response context and to the source text. Relations between the cohesion indices and reading skill were also somewhat stronger for reading comprehension as compared to vocabulary, potentially pointing to the importance of specific comprehension skills that elicit coherence-building amongst readers.

With respect to the second research question (To what extent do these relations vary across contexts (i.e., text and sample)?), we examined how the relations described above varied as a function of text genre (science, history) and across institutional contexts (community college, University). Our findings suggest that the relations between foundational reading skill and response cohesion were relatively stable across these contexts with some differences in magnitude. For students at a 2-year community college, cohesion measures were more strongly related to proficiency in reading skill than to vocabulary. By contrast, the students enrolled at the four-year institution showed an inverse effect, such that cohesion measures were more strongly correlated with vocabulary score as compared to reading comprehension proficiency. Although we caution over interpreting these modest effects, they may reflect the fact that community college and universities have different admissions criteria. Community colleges are open access and accept anyone who applies, whereas universities typically have admissions criteria based on performance on standardized tests (e.g., ACT, SAT). As such, there will likely be differences in the distribution of foundational skills of reading given that lower skilled readers will be admitted to community college than at universities. As such, there could be greater variability in foundational skills of reading in a community college setting, which underscores the challenges of providing specific support for college students who may be underprepared to read for their college coursework.

With respect to the stability across texts, we found similar relationships between the history and science texts used in this study. While there are disciplinary skills for comprehending and using history and science tests (Shanahan and Shanahan, 2008), there are also likely a common set of skills that support coherence building strategies. That is, readers need to accurately process words, activate relevant word and general knowledge, and use that knowledge to establish connections across content (Perfetti and Stafura, 2014). As such, variability in performance on these standardized tests are consistently correlated with computational indices of coherence building strategies. Given that there are only two texts in this data set, it is difficult to discern if the small differences across correlations reflect idiosyncratic variations that occur from text to text or if these differences point to more generalizable effects that might emerge in students reading of different genres or disciplines.

### Implications

These results have important theoretical implications. Theories of text comprehension emphasize the importance of coherence building strategies for comprehension but are arguably agnostic about the implications of individual differences in the foundational skills of reading on those strategies (McNamara and Magliano, 2009). On the other hand, theories of reading typically explicitly specify the relationships between foundational skills of reading and comprehension proficiency (e.g., Gough and Tunmer, 1986; Cromley and Azevedo, 2007). The results of the present study indicate that theoretical frameworks that describe how foundational skills of reading and coherence building strategies are coordinated are warranted (Perfetti and Stafura, 2014; Kopatich et al., 2018; Magliano et al., 2020). The results of the present study suggest that the computational analysis of constructed responses could be a valuable tool to test and refine these frameworks.

With respect to implications for practice, this study was motivated by the challenge of finding ways to learn about and help support early college students read for their coursework. This goal is motivated by the fact that many students come to college not ready to read (ACT, 2006; Baer et al., 2006; Holschuh and Paulson, 2013). While the computational analysis of constructed responses can be used to assess individual differences (e.g., Magliano et al., 2011), they are often used in computer-based interventions as a way to provide feedback to students (e.g., McNamara et al., 2004). Both the text comparison and the constructed response cohesion approaches assess the extent that readers are making connections between their thinking about the text and how their thoughts are grounded in the text. The computational approaches that we have used have been limited to assessing the global quality of constructed responses (e.g., McNamara et al., 2004). The results of this study suggest that computational approaches to examining constructed responses may be able to drive more specific feedback about students’ coherence-building processes when reading. For example, feedback could be given regarding the extent that students are grounding the text in the discourse context and establishing local and global coherence. The connections that students make could be visualized such that they can more easily see the connections they have made to the prior text content. Moreover, such analyses of constructed responses may help to reveal different profiles of strengths and challenges of readers (Rapp et al., 2007; McMaster et al., 2012; Kopatich et al., 2022). It is possible that some struggling college readers do not connect their thoughts to the text, whereas others show evidence of establishing local coherence, but need additional support learning strategies to establish global coherence. The present study was not designed to determine exactly how the indices explored in this study could be used in such applications, but it demonstrates that such research is warranted.

### Limitations and future directions

One limitation of this study is that we relied on a secondary data analysis and focused upon limited measures of foundational reading skill. Vocabulary and reading comprehension are but a few foundational reading skills that are thought to impact reading processes and outcomes. In the same vein, we selected a small set of theoretically-motivated linguistic indices related to dimensions of cohesion. These indices were selected to specifically target our dimensions of interest, but do not reflect all possible ways of capturing the cohesion of language. For example, the Tool for the Automatic Assessment of Cohesion (Crossley et al., 2016) provides more than 150 indices related to cohesion. Further, comprehension processes and individual differences are likely to manifest in other dimensions of language (e.g., lexical features, syntactic complexity) that were noted explored in this study. As such, it is important to acknowledge that the indices used in this study did not account for a large proportion of the variance in students’ vocabulary knowledge or comprehension proficiency. Our intent was not to fully model all of the proficiencies and processes related to reading comprehension (e.g., SEM) nor to produce an algorithm that can accurately predict readers’ individual differences, but rather to conduct a theoretically-driven exploratory analysis of a particular aspect of coherence-building (i.e., cohesion) as it might relate to foundational skills of reading.

However, the current work gives support to the need for more large-scale studies that “put together the pieces” of comprehension. As is core to the assumption of this work, reading involves complex, interactive processes that operate on multiple levels (Perfetti and Stafura, 2014). Thus, future work should include additional measures of foundational reading that can provide a more stable representation of the construct(s) in question (e.g., Cromley and Azevedo, 2007; Kopatich et al., 2018; Magliano et al., 2020) as well as additional relevant individual differences that are known to impact comprehension processes (e.g., prior knowledge; McCarthy and McNamara, 2021). Similarly, future work should extend the type and quantity of linguistic indices. Such studies would both increase our ability to model student learning through more accurate algorithms and through deeper understanding of the relations between student constructed responses and the underlying comprehension processes.

Additionally, there are significant challenges to support underprepared college readers (Calcagno and Long, 2008; Crisp and Delgado, 2014; Bailey et al., 2016; Ganga et al., 2018), finding ways to support college readers is of critical importance (Boylan and Trawick, 2015). There are dramatic changes to how students are provided support (Cormier and Bickerstaff, 2019). Traditionally, institutionally designated underprepared students have been required to pass literacy courses before they can take credit bearing courses (e.g., Bailey et al., 2016), and indeed a large portion of the sample in the present study were assigned to such a course. However, new approaches involve additional support while students take credit bearing courses. Tutoring systems could be developed specifically for struggling college readers that utilize constructed responses to provide individualized training to refine coherence building strategies. We believe the indices used in this study could be incorporated into such systems. However, more research is needed to develop these systems and to refine the computational algorithm that would be used to provide feedback.

A final issue to consider involves the manner in which the think aloud protocols were produced. Specifically, RSAT has participants type their thoughts, whereas thinking aloud typically involves orally producing thoughts (e.g., Ericsson and Simon, 1993). Prior research has shown that typed and orally produced think aloud protocols for college students are very similar in terms of the presence of comprehension strategies as identified by human judges (Muñoz et al., 2006). However, it is an open question as to whether the relationship between measures of coherence and comprehension outcomes and individual differences would be different if protocols were produced by typing or orally producing them. Speech to text software was not a viable data collection option when RSAT was created, whereas it is now. As such, it is important to assess how these measures might be affected by the modality in which think aloud protocols are produced.

## Conclusion

Our results indicate that the computational analysis of constructed responses produced by college readers has promise for theory and applications. Learning how to support underprepared college readers requires more research in the cognition of reading for understanding (Magliano et al., 2020; Perin, 2020). Constructed responses have promise in the context of this research. Moreover, they have promise in the context of intelligent tutoring systems that teach strategies to support reading for understanding (Graesser and McNamara, 2011). In this study we identified new approaches to analyze constructed responses produced during reading that will have important implications on the endeavors.

## Data availability statement

The original contributions presented in the study are included in the article/supplementary material, further inquiries can be directed to the corresponding author.

## Ethics statement

The studies involving human participants were reviewed and approved by University Research Services and Administration at Georgia State University. The patients/participants provided their written informed consent to participate in this study.

## Author contributions

JM was the lead investigator on the study, contributed to the conceptualization of the study, and was the lead author. LF was a co-investigator on the project, contributed to the conceptualization of the study and conducting the analyses, and was responsible for writing the results sections of the manuscript. DF was a co-investigator on the project and contributed to the conceptualization of the study and the writing of the manuscript. LA was a co-investigator on the project, contributed to the conceptualization of the study and was responsible for overseeing the analysis and the writing of the results section. KM was a co-investigator on the study, contributed to the conceptualization of the study and assisted in the writing of the manuscript. DM was a co-investigator on the study, contributed to the conceptualization of the study and assisted in the writing of the manuscript. All authors contributed to the article and approved the submitted version.
